# Transulnarversus transradial artery access in patients undergoing cardiac procedures: a systematic review and meta-analysis

**DOI:** 10.1097/MS9.0000000000004696

**Published:** 2026-01-21

**Authors:** Mohamed Falah Abdullah Al-Shamari, Sofian Mohamed M. Zreigh, Ahmed A. Mosa, Daniya Waqas, Yamin Htet, Iqra Iqbal, Efrah Ashraf, Sana Javaid, Imshal Farooq, Milikyas Abera Feyisa, Mohammad Ebad Ur Rehman, Huzaifa Ahmad Cheema, Asma’a Munasar Ali Alsubari, Abdulqadir J. Nashwan, Ameen M. Mohammad, Adeel Ahmad, Wajeeh Ur Rehman, Raheel Ahmed

**Affiliations:** a Department of Medicine, West Cumberland Hospital, Cumbria, UK; b Department of Medicine, Ankara Yildirim Beyazit University, Ankara, Turkey; c Department of Medicine, College of Medicine, University of Zakho, Zakho, Iraq; d Department of Medicine, Shaikh Khalifa bin Zayed Al Nahyan Medical and Dental College, Lahore, Pakistan; e Department of Medicine, University of Medicine, Yangon, Myanmar; f Department of Medicine, Services Institute of Medical Sciences, Lahore, Pakistan; g Department of Medicine, Bahawal Victoria Hospital, Bahawalpur, Pakistan; h Department of Medicine, Mayo Hospital, Lahore, Pakistan; i Department of Medicine, CMH Hospital, Lahore, Pakistan; j Department of Medicine, Naas General Hospital, Naas, Ireland; k Department of Medicine, Rawalpindi Medical University, Rawalpindi, Pakistan; l Department of Cardiology, King Edward Medical University, Lahore, Pakistan; m Faculty of Medicine, Sana’a University, Sana’a, Yemen; n Nursing & Midwifery Research Department, Hamad Medical Corporation, Doha, Qatar; o Department of Cardiovascular Disease, College of Medicine, University of Duhok, Duhok, Iraq; p Department of Cardiovascular Medicine, Mayo Clinic, Rochester, MN, USA; q Department of Internal Medicine, United Health Services Hospital, Johnson City, NY, USA; r Department of Cardiovascular Medicine, National Heart & Lung Institute, Imperial College London, London, UK; s Department of Cardiology, Royal Brompton Hospital, London, UK

**Keywords:** coronary angiography, percutaneous coronary intervention, radial artery, ulnar artery

## Abstract

**Background:**

Transradial artery access (TRA) is the favored approach for percutaneous coronary intervention (PCI) and coronary angiography (CAG). However, transulnar artery access (TUA) has emerged as an alternative, particularly when TRA is not feasible. This systematic review and meta-analysis aims to compare the efficacy and safety of TUA versus TRA in patients undergoing PCI and CAG.

**Methods:**

From inception to August 2025, a systematic search of electronic databases was conducted. Data were synthesized using a random-effects model, and heterogeneity was assessed using the *I*^2^ statistic.

**Results:**

Nine RCTs involving 6089 patients were included. Meta-analysis revealed no significant difference in procedural success between TUA and TRA (RR: 0.93, 95% CI: 0.82–1.06, *P* = 0.28, *I*^2^ = 99%). Similarly, puncture success rates were comparable (RR: 0.95, 95% CI: 0.91–1.00, *P* = 0.06, *I*^2^ = 83%). Secondary outcomes showed no significant differences in puncture attempts or hematoma incidence, but TRA was associated with a shorter procedure time (MD: 0.89, 95% CI: 0.27–1.50, *P* = 0.005).

**Conclusion:**

TUA provides a viable alternative to TRA, with similar success rates and complication profiles. However, TRA remains superior in terms of procedure time. Further large-scale RCTs are warranted to confirm these findings and refine clinical guidelines.

## Introduction

Percutaneous coronary intervention (PCI) and coronary angiography (CAG) are fundamental procedures in diagnosing and managing coronary artery disease (CAD), which results from the narrowing of coronary arteries due to atherosclerosis. PCI involves using a catheter to open blocked arteries, often with stents, to restore blood flow. CAG, on the other hand, is an imaging technique to visualize coronary arteries and identify blockages. These procedures play a crucial role in reducing the mortality and morbidity associated with CAD, a leading cause of death worldwide^[1^–^4]^. Cardiovascular diseases, including CAD, account for approximately 17.9 million deaths annually, representing 32% of all global deaths, according to the World Health Organization (WHO)^[5]^.

**HIGHLIGHTS**

- This meta-analysis included 9 RCTs and 6089 patients.
- Ulnar and radial artery access had similar success rates.
- Radial artery access had a shorter procedure time.

The prevalence of CAD increases with age and is more common in individuals with risk factors such as hypertension, diabetes, and smoking^[6]^. Traditionally, the femoral artery was the primary access point for PCI and CAG. However, in recent decades, trans-radial artery access (TRA) has gained prominence due to its association with fewer vascular complications, quicker recovery times, and greater patient comfort. TRA has become the preferred approach in many regions, particularly in Europe and North America. Nevertheless, TRA has its challenges, including a higher failure rate caused by vasospasm, smaller artery size, and anatomical variations^[7]^. Additionally, repeated use of the TRA can lead to intimal hyperplasia, complicating future interventions^[8]^.

The ulnar artery, an alternative forearm access route, has emerged as a viable option for patients where TRA is not feasible. Trans-ulnar artery access (TUA) offers potential advantages, including a larger artery diameter and, in some individuals, a more straightforward anatomical course. However, TUA is technically more challenging due to the deeper location of the ulnar artery and its proximity to the ulnar nerve, which increases the risk of nerve injury. Despite these challenges, the use of TUA has been explored in various studies, though with mixed results^[9,10]^.

For instance, a study by Hahalis *et al* found that TUA was a viable alternative to TRA, with comparable rates of procedural success and complications. This suggests that TUA could be more widely adopted^[11]^. However, contrasting findings from a study by Gralak-Lachowska *et al* (2020) showed higher rates of access failure and complications with TUA^[12]^, particularly among operators inexperienced with the technique. These conflicting findings have led to ongoing debates about the optimal access site for cardiac catheterization and intervention. Variability in outcomes may be partly due to differences in procedural guidelines across regions^[13]^. For example, the European Society of Cardiology^[14]^ guidelines favor TRA access for its established safety profile, while the American guidelines^[15]^ allow for more flexibility, considering patient and operator factors. The need to determine the most effective and safe access route for PCI and CAG, particularly in patients with complex anatomical considerations or where TRA is not feasible, underpins the rationale for comparing TUA and TRA. This systematic review and meta-analysis aim to synthesize existing randomized controlled trials (RCTs) comparing these two access methods, focusing on procedural success rates, complication rates, and overall safety profiles. Our objective complements broader comparative syntheses by restricting inclusion to head-to-head randomized trials of transulnar versus transradial access in coronary angiography and PCI, while extracting technical factors such as ultrasound guidance and introducer sheath size to improve the directness and interpretability of estimates.

## Materials and methods

This meta-analysis has been conducted in accordance with the comprehensive guidelines outlined in the Cochrane Handbook for Systematic Reviews of Interventions. Furthermore, the reporting of our findings follows the standards set by the Preferred Reporting Items for Systematic Reviews and Meta-Analyses (PRISMA) statement^[16]^. Our study protocol has also been registered with PROSPERO. Ethical approval was not required for this manuscript.

### Data source and searches

A systematic and extensive electronic search was undertaken in ClinicalTrials.gov, MEDLINE, Embase, and the Cochrane Central Register of Controlled Trials from their inception through to August 2025. Additionally, reference lists from included studies and related systematic reviews were carefully examined to identify any further relevant studies. Medical Subject Heading (MeSH) terms and keywords for ulnar artery, radial artery, percutaneous coronary intervention and coronary angiography were used.

### Eligibility criteria

This meta-analysis included all RCTs that directly compared TUA with TRA access in patients undergoing PCI or CAG. Exclusion criteria were study designs other than RCTs, such as quasi-randomized trials, cohort or case-control studies, cross-sectional studies, and any research conducted on animal models. Additionally, studies lacking a direct comparison between TUA and TRA were excluded. No restrictions were applied regarding the language of publication or the date of publication.

### Study selection and data extraction

We used Rayyan software to manage the search results and remove duplicates effectively. Two authors independently screened the titles and abstracts to ensure that only relevant studies were retained. The remaining studies underwent full-text screening based on our eligibility criteria. A third author resolved any disagreements between the reviewers.

Relevant data were extracted into a pre-piloted Excel spreadsheet, capturing key characteristics such as the reviewer’s name, authors’ names, publication year, country of origin, sample size, mean age (with standard deviation), gender distribution, and the prevalence of comorbidities, including hypertension, diabetes, dyslipidemia, and smoking habits. Additionally, data on prior medical history, including myocardial infarction (MI) and stroke, were recorded. The type of cardiac procedure performed (PCI/CAG), median follow-up duration, details of the intervention, and outcomes were collected. Where reported, we extracted whether vascular access was obtained with ultrasound guidance and the introducer sheath size in French units, recognizing that incomplete reporting would limit stratified analyses. We also extracted the occurrence of ulnar nerve injury when reported and coded studies as not reported when this outcome was absent.

### Outcomes

The primary outcomes of our study were procedural success rate and puncture success rate. Secondary outcomes included the number of puncture attempts, procedure time, and complications such as hematoma and arterial spasm. Outcome definitions were prespecified as follows. Procedural success was completion of the intended diagnostic or interventional procedure via the assigned forearm artery without crossover. Access success was defined as successful sheath insertion with advancement of the diagnostic guide wire via the assigned artery. Hematoma followed the study specific grading, and when grading scales differed we pooled any hematoma. Spasm was defined as angiographic or clinically evident spasm requiring treatment or materially impeding catheter manipulation, according to each trial’s definition. Ulnar nerve injury encompassed any new sensory or motor deficit or neuropathic symptoms attributable to vascular access.

### Risk of bias assessment

The risk of bias in the included studies was assessed using the revised Cochrane Risk of Bias tool for randomized trials (RoB 2.0)^[17]^. This tool evaluates potential bias across five key domains: (1) bias arising from the randomization process; (2) bias due to deviations from the intended interventions; (3) bias resulting from missing outcome data; (4) bias in the measurement of outcomes; and (5) bias in the selection of reported results. Each study’s risk of bias was independently assessed by two investigators, with ratings classified as high, low, or raising some concerns of bias. Any disagreements between the investigators were resolved through consultation with a senior investigator.

### Data synthesis

To conduct the meta-analyses, we utilized Review Manager (RevMan, Version 5.4.1) software. The Mantel-Haenszel method was employed for dichotomous outcomes, with risk ratios (RRs) and their corresponding 95% confidence intervals (CIs) extracted. For continuous outcomes, the inverse variance method was used to pool mean differences (M.D.) along with their 95% CIs. A random-effects model was applied to account for potential variability across studies. Pooled estimates were presented in forest plots, and statistical heterogeneity was assessed using the Higgins I-square (*I*^2^) statistic. Where at least ten studies contributed to an outcome, small-study effects and publication bias were to be explored using funnel plots and regression-based tests; when fewer than ten studies were available, such analyses were not performed due to low reliability.

## Results

### Search results

**Figure 1.**
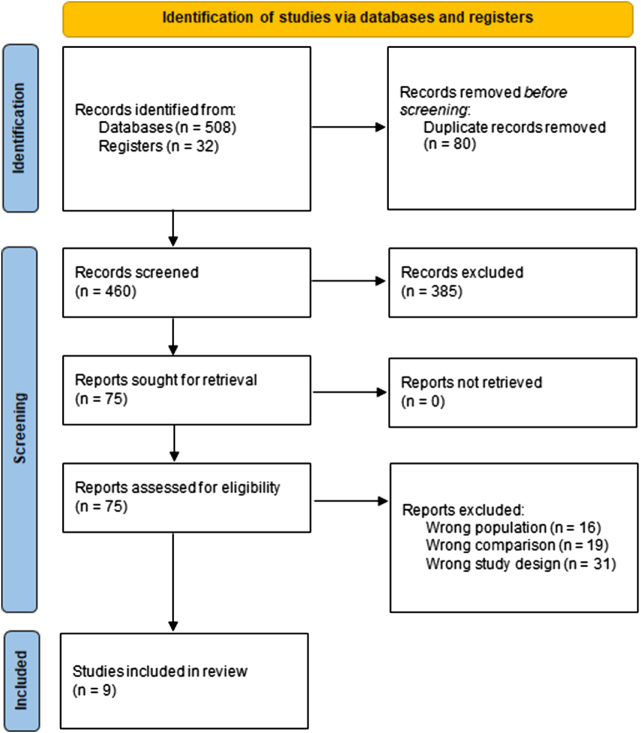
PRISMA flowchart.

The initial search yielded 540 articles from databases and registers. After removing 80 duplicate records, 460 records were screened based on titles and abstracts, leading to the exclusion of 385 articles. Full-text screening of the remaining 75 reports resulted in the exclusion of 66 studies. Ultimately, nine studies were included in this meta-analysis. The detailed screening process is illustrated in Figure 1.

### Study characteristics

This meta-analysis included nine RCTs^[11,18–25]^ conducted across five countries (France, China, Egypt, India, and Greece) with a total of 6089 patients undergoing PCI or CAG. The publication years ranged from 2006 to 2024, with sample sizes ranging from 150 to 2532 patients. Follow-up durations spanned from 1 week to 12 months. Key study characteristics are detailed in Table 1. Where available, the use of ultrasound guidance and the introducer sheath size are also presented, and denoted as not reported when absent. Reporting of these variables was inconsistent across trials. Relation to prior evidence and contribution: Recent comparative syntheses, including network meta-analyses spanning femoral, proximal and distal radial, and ulnar access^[26]^, have reported broadly similar procedural outcomes across upper-extremity strategies and continue to support radial access in contemporary practice. The present review addresses a different question by providing direct, randomized, head-to-head estimates for transulnar versus transradial access specifically in coronary angiography and PCI, updated through August 2024. In doing so, it also highlights persistent reporting gaps for technical modifiers such as ultrasound guidance and introducer sheath size and the sparse, inconsistent ascertainment of ulnar nerve injury. The distal versus proximal radial comparison remains important but lies outside the predefined scope of this analysis.

**Table 1:** Characteristics of included studies

Study ID	Country	Sample size	Mean age, in years (SD)	Male (%)	Hypertension (%)	Diabetes (%)	Smoking (%)	Dyslipidemia (%)	Prior MI (%)	Prior stroke (%)	Type of procedure	Ultrasound guidance (Y/N/NR)	Sheath size (Fr)	Median follow-up
Aptecar 2006	France	431 (216 vs 215)	63 (12) vs 63 (13)	73.5	NR	21.65	47.3	NR	NR	NR	PCI/CAG	NR	NR	1 month
Bi 2017	China	445 (220 vs 225)	58.2 (7.3) vs 57.6 (6.1)	65.84	51.24	25.62	40.67	27.19	NR	NR	PCI/CAG	NR	NR	12 months
Elwany 2024	Egypt	150 (50 vs 100)	59.26 (8.92) vs 58.33 (7.87)	72	65.3	50	56.7	NR	NR	NR	PCI/CAG	Y	6 Fr	28 days
Geng 2014	China	535 (271 vs 264)	64.2 (10.1) vs 65.4 (9.4)	67	65	27	32	51	9	9	PCI/CAG	NR	NR	1 month
Gokhroo 2016	India	2532 (1270 vs 1262)	67.1 (11.4) vs 63.1 (12.1)	62	44	34	39	23	NR	NR	PCI	NR	5–6 Fr	1 week
Hahalis 2013	Greece	902 (462 vs 440)	64.3 (10.8) vs 64.6 (11.9)	78	59	28	44	23	17	NR	PCI/CAG	NR	6 Fr	60 days
Li 2010	China	240 (118 vs 122)	60 (10) vs 61 (10)	67	65	35	44	45	7	9	PCI/CAG	NR	6 Fr	30 days
Liu 2014	China	636 (317 vs 319)	58.6 (11.5) vs 59.2 (11.4)	68	46	20	47	18	NR	NR	PCI	Y	6 Fr	1 year
Ranwa 2019	India	218 (108 vs 110)	53.8 (9.58) vs 55.32 (9.87)	81.2	34	32.1	24.3	25.2	NR	NR	PCI	NR	NR	1 week

CAG, coronary angiography; MI, myocardial infarction; NR, not reported; PCI, percutaneous coronary intervention; SD, standard deviation.

Ultrasound guidance and sheath size were extracted from trial reports where available. “Y” indicates ultrasound guidance used; “N” indicates not used; “NR” indicates not reported. Sheath sizes are shown in French (Fr) units. Where sheath ranges (e.g., 5–6 Fr) were reported, both values are listed.

### Risk of bias

The quality assessment of the included studies is summarized in Supplemental Digital Content Figure 1, available at: http://links.lww.com/MS9/B73. Out of the nine studies, two were assessed as having a low risk of bias, while three studies were identified as having a high risk of bias. The high risk was primarily due to issues related to randomization, outcome measurement, and selection of reported results. Four studies exhibited some concerns regarding bias, with the main issues stemming from deviations from the intended interventions, randomization processes, and the measurement of outcomes.

### Procedural Success Rate

#### Puncture attempts

**Figure 2.**
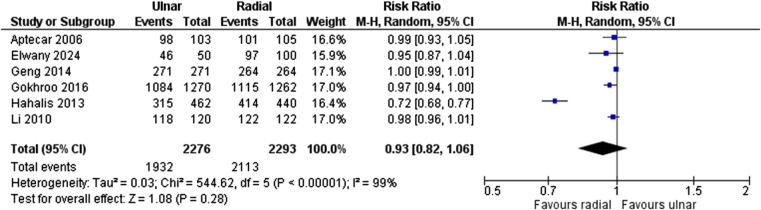
Forest plot for procedure success.

Six studies were included in the analysis of procedural success rate, involving 4569 patients (2276 TUA vs 2293 TRA). The analysis did not reveal a significant difference between TUA and TRA in terms of procedural success (RR: 0.93, 95% CI: 0.82–1.06, *P* = 0.28, *I*^2^ = 99%; Fig. 2).

#### Puncture Success Rate

**Figure 3.**
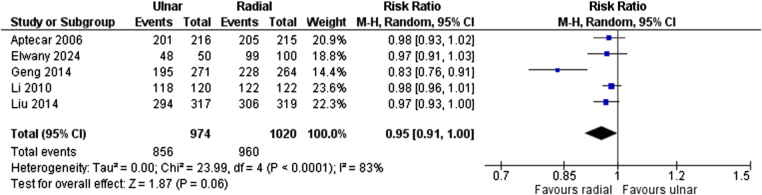
Forest plot for puncture success.

The puncture success rate was assessed through the synthesis of data from five studies involving 1994 patients, of whom 974 were assigned to TUA and 1020 to TRA. The findings demonstrated no significant difference in puncture success between the two groups (RR = 0.95, 95% CI: 0.91–1.00, *P* = 0.06, *I*^2^ = 83%; Fig. 3).

### Meta-analysis of secondary outcomes

#### Puncture attempts

Six studies, including 2478 patients (1227 TUA vs 1251 TRA), assessed the number of puncture attempts. The analysis revealed no significant difference between TUA and TRA (MD: 0.47, 95% CI: − 0.05 to 1.00, *P* = 0.08, *I*^2^ = 98%; Supplemental Digital Content Figure 2, available at:http://links.lww.com/MS9/B73).

#### Procedure time

Seven studies, including 3559 patients (1764 TUA vs 1795 TRA), reported on procedure time. The results demonstrated that TRA was associated with a significantly shorter procedure time compared to TUA (MD: 0.89, 95% CI: 0.27 to 1.50, *P* = 0.005, *I*^2^ = 88%; Supplemental Digital Content Figure 3, available at: http://links.lww.com/MS9/B73).

#### Hematoma

Eight studies, with a total of 5314 patients (2641 TUA vs 2673 TRA), evaluated the incidence of hematoma. The analysis showed no statistically significant difference between the two access routes (RR: 0.85, 95% CI: 0.57 to 1.27, *P* = 0.43, *I*^2^ = 58%; Supplemental Digital Content Figure 4, available at: http://links.lww.com/MS9/B73).

#### Spasm

Nine studies involving 5834 patients (2906 TUA vs 2928 TRA) assessed the incidence of arterial spasm. No significant difference was found between the two groups (RR: 1.29, 95% CI: 0.96 to 1.73, *P* = 0.09, *I*^2^ = 0%; Supplemental Digital Content Figure 5, available at: http://links.lww.com/MS9/B73). For most outcomes, fewer than ten studies contributed data, which limited the interpretability of funnel plots and formal tests for small-study effects; therefore, publication bias cannot be excluded. Ulnar nerve injury was infrequently reported and was not amenable to quantitative synthesis, so available events are reported descriptively where provided.

## Discussion

Our analysis of nine RCTs involving 6089 participants found no significant differences in procedural success and puncture success between TUA and TRA during coronary CAG or PCI. Nonetheless, between-study heterogeneity was substantial across several endpoints, including procedural success where *I*^2^ reached 99 percent, and therefore pooled estimates should be interpreted with caution. This variability likely reflects differences in operator experience and learning curve, variation in patient selection between elective CAG and PCI including acute presentations, and technical factors such as use of ultrasound guidance versus palpation, sheath size selection, and center volume. The deeper course and anatomical variability of the ulnar artery may heighten the influence of these factors relative to the radial approach. A random-effects model was prespecified to account for anticipated clinical and methodological diversity. However, incomplete and non-uniform reporting of these potential effect modifiers precluded robust subgroup or meta-regression analyses, so emphasis is placed on the direction and consistency of effects rather than the precise magnitude of pooled estimates. Ultrasound guidance may mitigate several technical challenges inherent to transulnar access by enabling real-time visualization of vessel depth, diameter, and course, optimizing puncture trajectory, and avoiding adjacent neurovascular structures. Where used, ultrasound guidance is expected to improve first-pass success, reduce puncture attempts and crossovers, and potentially decrease vasospasm and access-site hematoma, particularly in patients with deeper or smaller-calibre ulnar arteries. In the present evidence base, reporting of ultrasound use was inconsistent and often absent, which precluded formal stratification or meta-regression by guidance modality. We therefore report ultrasound use descriptively where available and recommend standardized reporting and prespecified use of ultrasound guidance in future randomized trials. Introducer sheath size may also modulate outcomes, with smaller systems such as 4 Fr and contemporary thin-walled 5 Fr sheaths plausibly reducing spasm and access-site trauma at the expense of device compatibility for complex PCI, whereas 6 Fr systems facilitate more demanding PCI at a potential cost of increased vasoreactivity and tissue injury; because sheath sizes were not uniformly reported, stratified analyses were not feasible.

These findings are consistent with a broader body of research, demonstrating that TUA is non-inferior to TRA in various procedural outcomes. For instance, Dahal *et al* conducted a meta-analysis that demonstrated that while TUA had a higher crossover rate due to technical challenges, it was equally effective as TRA in terms of major adverse cardiovascular events and access-related complications^[27]^. Similarly, Sedhom *et al* performed a systematic review and meta-analysis, which analyzed data from 5721 patients across seven RCTs and found no significant differences between TUA and TRA approaches in terms of procedural success and complication rates^[28]^. Comparably, Kar *et al* conducted a study comparing ultrasound-guided TUA and TRA. Their results revealed no significant difference in puncture success rates between TUA (89.8%) and TRA (91.3%). This study underscores that with proper ultrasound guidance, TUA can achieve puncture success rates comparable to TRA, supporting its utility in clinical practice^[29]^. In a related study conducted at an Egyptian cardiology center, Shafiq *et al*^[30]^ also reported no significant differences in procedural outcomes between the TUA and TRA approaches, including PCI success rates (*P* = 0.699). This study further demonstrated comparable complication rates, including the incidence of hematoma and arterial spasm, between the two groups^[30]^.

Our meta-analysis demonstrates comparable access-related complications between both groups. Consistent with our findings, a prior meta-analysis by Sedhom *et al* found no significant differences in access-site complications between TUA and TRA. Additionally, the risk of vasospasm was similar between the two groups. Interestingly, the study noted a lower risk of local bleeding in the TUA group compared with TRA, suggesting a potential advantage of TUA in reducing bleeding complications^[28]^.

The challenges associated with TUA during coronary CAG and PCI stem from its anatomical and physiological characteristics, which contribute to higher crossover rates compared to TRA. Shafiq *et al*^[30]^ demonstrated that TUA had a significantly higher crossover rate due to increased tortuosity and deeper anatomical positioning^[27]^. This is further complicated by the ulnar artery’s proximity to the ulnar nerve, raising the risk of complications such as nerve injury and spasms during puncture^[31–33]^. Moreover, endothelial cells lining the ulnar artery may release less nitric oxide (NO) during vascular injury, potentially exacerbating the risk of vascular spasms^[34–36]^. The ulnar artery’s extracellular matrix (ECM) composition, which may include a different ratio of collagen and elastin compared to the TRA, could further contribute to its susceptibility to injury during puncture, particularly in the context of the artery’s deeper location and increased tortuosity^[37–39]^. Additionally, the role of transforming growth factor-beta (TGF-β) in vascular remodeling is crucial; this cytokine influences the behavior of vascular smooth muscle cells and ECM remodeling, impacting the artery’s response to mechanical stress and injury^[40,41]^.

The analysis of secondary outcomes revealed no significant differences in puncture attempts between TUA and TRA. However, procedure time was significantly shorter for TRA. These findings align with other systematic reviews that reported similar trends. For instance, El- Shafiq *et al*^[30]^ found no significant differences in puncture attempts between the two access routes (*P* > 0.05), but they noted that procedure time was shorter for TRA^[30]^. Similarly, Hahalis *et al* found TRA to be associated with shorter procedure times, primarily due to its superficial anatomical location, which facilitates easier catheter manipulation^[11]^. The high heterogeneity (*I*^2^ = 98%) observed in puncture attempts suggests that factors such as operator experience, procedural technique, and patient anatomy may contribute significantly to the variability in results. A meta-analysis by Sedhom *et al* supports this finding, indicating that despite comparable success rates, the deeper anatomical location of the ulnar artery and its increased tortuosity can make it more challenging to access, leading to higher crossover rates that emphasize the importance of operator experience in determining access success^[28]^.

The shorter procedure time associated with TRA is likely due to its anatomical advantages. Studies such as Hahalis *et al* also reported shorter procedure times for TRA, with similar findings of reduced procedural duration due to the radial artery’s more superficial location and easier accessibility^[11]^. These results underscore the importance of operator expertise and the use of ultrasound guidance to mitigate challenges associated with TUA^[28]^. A meta-analysis by Vidovich^[42]^ and Scalise *et al*^[43]^ support this finding this finding, highlighting that despite comparable puncture success rates between TUA and TRA, the deeper anatomical location and increased tortuosity of the ulnar artery contribute to more technical difficulties and higher crossover rates from TUA to TRA^[42,43]^. Another study by Hahalis *et al* corroborates these results, showing that TRA, due to its more superficial and easily accessible location, resulted in shorter procedure times and fewer puncture attempts compared to TUA^[11]^.

TRA remains preferred due to its shorter procedure time and easier manipulation, but TUA is a valuable alternative when TRA is not feasible, particularly in patients with anatomical variations or prior interventions. Both access routes offer advantages over femoral access, particularly in reducing bleeding complications, and the choice should ultimately be based on patient-specific factors and operator expertise^[44–47]^.

Our study has several limitations. Firstly, a majority of the included studies were single-center trials with relatively small sample sizes, which limits the generalizability of the findings. Multicenter studies with larger populations are needed to validate these results. Secondly, there was variability in operator experience, particularly with TUA, which could have influenced the outcomes, as less experienced operators may not have achieved similar success rates as those seen in the studies. Thirdly, none of the studies included a long-term follow-up, making it difficult to assess the durability of the outcomes, particularly regarding vascular complications such as arterial occlusion and late hematomas. Additionally, several studies excluded patients with challenging anatomical features or those requiring primary PCI, which limits the applicability of the results to all patient populations. Finally, the open-label design of many trials might have introduced bias, particularly in the subjective assessment of procedural success and complications. Further research with blinded assessments and standardized protocols is warranted to address these limitations. In addition, the small number of contributing studies for several endpoints precluded robust evaluation of small-study effects and publication bias, and as such the presence of publication bias cannot be excluded. Furthermore, ulnar nerve injury was rarely prespecified or systematically assessed, and inconsistent reporting across trials precluded meta-analysis, leaving the true incidence uncertain. Finally, our focus on head-to-head transulnar versus transradial trials means that questions specific to radial subsite selection, such as distal versus proximal radial access, were not evaluated here.

## Conclusion

This meta-analysis concludes that TUA offers a comparable alternative to TRA access for PCI or CAG without significantly affecting procedural success rates, puncture success, or secondary outcomes such as hematoma, spasm, and procedure time. However, given the moderate heterogeneity and potential biases in some studies, these findings should be interpreted with caution. Further well-designed, large-scale RCTs are needed to confirm the safety and efficacy of TUA before it can be recommended as a routine clinical practice.

## Data Availability

The data supporting this study’s findings are available from the corresponding author upon reasonable request.

## Research registration unique identifying number (UIN)

CRD42024585614.
